# Current Aspects of the Endocannabinoid System and Targeted THC and CBD Phytocannabinoids as Potential Therapeutics for Parkinson’s and Alzheimer’s Diseases: a Review

**DOI:** 10.1007/s12035-020-02054-6

**Published:** 2020-08-19

**Authors:** R. Cooray, V. Gupta, C. Suphioglu

**Affiliations:** 1 Faculty of Science, Engineering and Built Environment, School of Life and Environmental Sciences, Deakin University, Waurn Ponds, Victoria 3216 Australia; 2 Faculty of Health, School of Medicine, Deakin University, Waurn Ponds, Victoria 3216 Australia; 3 Section of Genetics, Institute for Research & Development in Health & Social Care, Colombo, Sri Lanka

**Keywords:** Parkinson’s disease, Alzheimer’s disease, Endocannabinoid system, THC, CBD

## Abstract

Neurodegeneration leading to Parkinson’s disease (PD) and Alzheimer’s disease (AD) has become a major health burden globally. Current treatments mainly target controlling symptoms and there are no therapeutics available in clinical practice to preventing the neurodegeneration or inducing neuronal repairing. Thus, the demand of novel research for the two disorders is imperative. This literature review aims to provide a collection of published work on PD and AD and current uses of endocannabinoid system (ECS) as a potential drug target for neurodegeneration. PD is frequently treated with l-DOPA and deep brain stimulation. Recent gene modification and remodelling techniques, such as CRISPR through human embryonic stem cells and induced pluripotent stem cells, have shown promising strategy for personalised medicine. AD characterised by extracellular deposits of amyloid β-senile plaques and neurofibrillary tangles of tau protein commonly uses choline acetyltransferase enhancers as therapeutics. The ECS is currently being studied as PD and AD drug targets where overexpression of ECS receptors exerted neuroprotection against PD and reduced neuroinflammation in AD. The delta-9-tetrahydrocannabinoid (Δ^9^-THC) and cannabidiol (CBD) cannabinoids of plant *Cannabis sativa* have shown neuroprotection upon PD and AD animal models yet triggered toxic effects on patients when administered directly. Therefore, understanding the precise molecular cascade following cannabinoid treatment is suggested, focusing especially on gene expression to identify drug targets for preventing and repairing neurodegeneration.

Neurodegenerative disorders (NDDs), characterised by progressive atrophy of nerve cells in both central and peripheral nervous systems, have become a major disease burden not only in low- and middle-income countries (LMICs) but also in the developed world as well. Among all neurodegenerative disorders, 1.8% account for Parkinson’s disease (PD) while Alzheimer’s disease (AD) accounts for 12% with the current rate reporting disease incidences are higher in LMICs [1]. Thus, the demand for novel research for the two disorders is imperative.

With the emerging studies on determining the pathophysiology of neurodegenerative disorders, the endocannabinoid system (ECS) has drawn a significant attention among researchers due to its potential neuroprotective effects. In this review, the authors aim to provide a collection of published literature of current use of the ECS and its components as a drug target for the most common NDDs, namely PD and AD. The review also discusses the use of external cannabinoids, mainly the THC and CBD, to target ECS in developing therapeutics for the two diseases.

## Parkinson’s Disease: Current Treatments and Advancements

Parkinson’s disease (PD) is the most prevalent motor disease and second most prevalent NDD [2], characterised by progressive death of dopaminergic neurons, predominantly in substantia nigra pars compacta (SNc), and in neostriatum, and subthalamic nucleus of basal ganglia at low amounts [3]. The intracellular accumulation of Lewy bodies enriched in α-synuclein protein, and thread-like proteinaceous inclusions called Lewy neuritis, is known to provoke the motor symptoms in PD [4]. The genetic studies describe that the mutations in α-synuclein [5, 6], *PINK1* [7], DJ-1 (*PARK7*) [8, 9], ubiquitin genes such as ubiquitin-C-hydrolase [10, 11], and population-specific gene mutations such as glucocerebrosidase gene in Ashkenazi Jews [12] directly relate to the onset of PD [13]. Evidence suggests that mutations in molecular signalling such as leucine-rich repeat kinase 2 (LRRK2) [14] and Miro GTPases [15] play a vital role in the onset and the progression of PD.

In addition to the l-DOPA, the most commonly used drug for PD, other physiological methods such as deep brain stimulation (DBS) with the basis of Network Centric Therapy are used to control the motor symptoms of PD, such as tremors and stiffness [16]. Furthermore, in attempting hormone therapy, Bourque and colleagues recently reviewed the potential of steroids, especially the sex steroids such as oestrogen and progesterone to be developed as therapy for PD via neurotransmitter modulation of selective brain oestrogen receptors [17]. However, as the authors suggest, an optimised formula should be developed with currently available drugs to specifically target brain receptors. Furthermore, an intervention study by a group of Japanese scientists recently utilised the gene modification and remodelling techniques such as CRISPR through human embryonic stem cells (hESCs) and induced pluripotent stem cells (iPSCs) which showed promising strategy for personalised medicine [18]. The idea, however, requires intensive investigations at a clinical level with a large cohort. The concept of using stem cells has been successfully tested in PD monkey models [19]; however, some level of ethical concern arose when obtaining hESCs at larger amounts for clinical trials. This limitation is addressed by a group of European researchers who initiated a global programme in developing cell-based therapies for PD [20]. The team highlights the challenges to face when developing the stem cell–based therapy such as identifying the most suitable cell line, development of clinical administrable cell product, quality control and quality assurance, pre-clinical testing for accurate dosing, and other measures [20].

Considering the circumstances, further interventions are essential in order to achieve a precise level of control in progression of dopaminergic neurodegeneration. However, to date, no drug has been identified to cease the progression of the neurodegeneration in PD.

## Alzheimer’s Disease: Current Treatments and Advancements

As the leading cause of dementia, and the most prevalent NDD, Alzheimer’s disease (AD) is characterised by extracellular deposits of amyloid β-senile plaques and intracellular neurofibrillary tangles composed of hyperphosphorylated tau protein [21], and reduced levels of choline acetyltransferase [22, 23]. The involvement of –omics in the onset of AD has been popular during the last few years. Four most common genetic mutations responsible for AD onset have been identified as follows: missense mutation in β-amyloid precursor protein (APP), *ApoE* allele mutations, and Presenilin 1 and 2 (PS1&2) [21]. However, with the recent developments of genome-wide association studies (GWAS), the determination of single-nucleotide polymorphism (SNP) variations has been possible at numerous locations simultaneously, with higher statistical power. Thus, implementing GWAS methods has uncovered several potential genetic mutations, which may not only be responsible for the onset of AD but also are heritable, with the use of epigenetic studies [24]. Further to the development of bioinformatics tools, analysis of differential gene expression has been attempted for protein-coding and non-coding RNA for AD brain [25]. The study has identified 18 non-coding and 7 protein-coding RNAs in brain tissues and thus their potential of using as a diagnostic marker for AD. Therefore, further transcriptomic studies can be suggested to provide a promising strategy to determine the risk of AD for penetrative measures and potentially to cease neurodegeneration.

A recent publication reviewed the association of metabolomics in the onset of AD as it gave an impression on genetics, transcriptomics, proteomics, and environmental factors of the disease [26]. The brain consumes the largest proportion of metabolised glucose for energy generation. However, metabolic impairment and consequence reduction of glucose uptake are commonly seen in AD [26]. All these strategies can immensely be used in the development of novel therapeutics for controlling AD.

Microglia, as the resident immune cells in the central nervous system, is known to play a critical role in brain homeostasis. Being one of the major pathological causes of neurodegeneration, neuro-inflammation induced through microgliopathy has been a popular research theme. Microglia dysfunction leading to neuroinflammation, commonly known as microgliopathy, is a common type of gliosis, associated with several genetic mutations including triggering receptor expressed on myeloid cells-2 (TREM-2). The importance of TREM-2 and related microgliopathy has been widely discussed in several recent reviews due to its significant association with neurodegenerative disorders, primarily in AD [27, 28]. The complete knock-out of *TREM-2* in AD mice models showed affected microglial activation decreasing the amount of microglia around the plaques [29]; however, the precise mechanism is yet to be understood. Collectively, research evidence the wide role of TREM-2 in reducing microglial cell proliferation [30], microglial survival, increasing apoptosis [31], and microglial autophagy [28, 32]. In addition to *TREM-2*, the role of other microglia genes such as *CR1*, *SPI1*, the *MS4As*, *ABCA7*, *CD33*, and *INPP5D* has been reviewed [33]. The understanding of these microglial genetics in depth as AD risk factors would provide an insight into disease mechanism in deriving therapeutics.

Most common treatments for AD include inhibitors of acetylcholinesterase (AChE) activity [34, 35] such as donepezil, rivastigmine, galantamine [36], and N-methyl-d-aspartate (NMDA) receptor antagonists [37] in the brain. Modulations of the immune system via targeting the specific immune modulators as well as their related genes have been popular in recently published literature [38]. As evidenced in a recent study, the characteristic deprived-expression of toxic pro-inflammatory M1 microglia genes, CD11b, iNOS, COX-2, and IL1β, in AD was restored by using low doses of curcumin in APPsw transgenic mice [39] thus addressing the anti-inflammatory property of curcumin on microglia. Recently, using a high dose of monoclonal antibodies targeting β-amyloid (Aβ) plaques has been introduced as a therapy [40]. However, these therapeutic advances so far target the control of symptoms, and as yet, no long-term resolution for neurodegeneration or prevention effect of the disease is proven.

## The Endocannabinoid System

Among the many molecular pathways involved in PD and AD pathophysiology, the endocannabinoid system (ECS) has drawn a significant attention in the past decade. With the knowledge that external cannabinoid compounds such as extracted cannabinoids (also known as phytocannabinoids) from the plant *Cannabis sativa* can act on the brain endocannabinoid system, the development of novel ECS-targeted therapeutics has become a popular theme.

The ECS of the central nervous system plays several regulatory functions including cognition, appetite control, and analgesia [41]. The endogenous cannabinoids are presumed to mediate neuronal plasticity via regulation of potentiation, inhibition, and disinhibition of synaptic output, ultimately modulating synaptic functions [42]. Although the exact molecular mechanism is not elucidated, further research will aid our understanding of complex processes involved, including cognitive functions, learning, and memory.

The ECS consists mainly of two primary receptors of family G protein–coupled receptors (GPCRs): cannabinoid receptors 1 and 2 (CB1R and CB2R), and two primary endogenous cannabinoid (endocannabinoid) neurotransmitters: *N*-arachidonoyl ethanolamine (AEA), i.e. anandamide [43] and 2-arachidonoyl glycerol (2-AG) [44, 45].

Anandamide is mainly synthesised by catalysing N-acyl-phosphatidylethanolamine (NAPE) by the catalysing enzyme NAPE-specific phospholipase D (NAPE-PLD) [46] while 2-AG is synthesised from diacylglycerol (DAG) by DAG lipase (DAGL) -α or -β types, mostly by -α type in adult human brain [47, 48]. The main enzyme responsible for anandamide degradation is the integral membrane protein fatty acid amide hydrolase (FAAH) which hydrolyses anandamides into free arachidonic acid and ethanolamine [49]. In addition to FAAH [50], 2-AG is mainly hydrolysed by three enzymes in the α/β hydrolase superfamily, monoacylglycerol lipase (MAGL), α/β-hydrolase domain containing 6 (ABHD6) and 12 (ABHD12) [51], to produce arachidonic acid and glycerol [52]. Among the three enzymes, MAGL is well characterised for its structure and function, yet the ABHD6 and ABHD12 remain uncharacterised at molecular level. In addition to enzymatic degradation, they can be degraded by oxidation through cyclooxygenase-2 and several lipoxygenases [47, 48, 53, 54].

The CB1R, encoded by the gene *CNR1*, consists of 472 amino acids in humans and found in three different isoforms: one canonical long form (dominates in the brain and skeletal muscles) and two with 33 amino acid deletion at N-terminal (dominant in liver and pancreatic islet cells, i.e. involves in metabolism), whereas CB2R is encoded by the gene *CNR2* and consists of 360 amino acids in humans, with only 44% sequence similarity with CB1R [55]. Among the two isoforms of CB2R identified to the date, one is dominant in the testis and lower levels in brain reward regions, and the other in the spleen and at lower levels in the brain [56]. In addition, CB2R is also observed to express in peripheral tissues such as leukocytes [56] and dendritic cells [57]. Apart from the aforementioned receptors, the endocannabinoids are known to act on non-CB1/CB2 GPCRs. The G protein–coupled receptor 55 (GPR55) expressed in dorsal root ganglion neurons is activated by endocannabinoids such as anandamides as well as phytocannabinoids like as Δ^9^-THC, and upon activation, may enhance intra-cellular Ca^2+^ levels [58].

The ECS is evidenced to follow a retrograde signalling, where the endocannabinoids are released from the post-synaptic end and receptors are localised to pre-synapses [48, 59]. The endocannabinoid release can be regulated by calcium influx or calcium-independent pathways [60]. In brief, 2-AG is synthesised in post-synapse and, being lipid messenger, is easily diffused through the membrane into the synaptic cleft towards the pre-synapse in a mechanism which is yet to be elucidated. It activates CB1R in pre-synapse to block signalling neurotransmitter release through suppression of Ca^2+^ influx via inhibiting voltage-gated Ca^2+^ channels or by inhibiting cAMP/PKA pathway which inhibits adenylyl cyclase. The 2-AG also identified to activate CB1R receptors in astrocytes [61]. The cannabinoid signalling is ceased via degradation of 2-AG by monoacylglycerol lipase (MAGL) [48]. Anandamides, on the other hand, diffuse into the synaptic cleft and activate CB1R, or other non-CBRs such as transient receptor potential cation channel subfamily V member 1 (TRPV1) as a full agonist. Anandamides are also synthesised in pre-synapse yet it is not clear whether they are responsible for anterograde signalling process [48].

The ECS and its components have been widely studied in search for therapeutics for PD and AD [62, 63]. The co-expression of CB1R and GPR55 has shown a significant neuroprotection against parkinsonism-inducing MPP^+^ toxin [64]. A similar study was conducted using PC12 cell lines with 6-hydroxydopamine (6-OHDA) neurotoxin–induced PD model treated with anandamide and observed anandamide-mediated neuroprotection through preventing apoptosis [65]. Moreover, similar to the effect of anandamide, the 2-AG endocannabinoids have shown neuroprotection against parkinsonism-inducing neurotoxin to prevent cellular apoptosis [66]. Furthermore, anandamide treated in combined noladin ether (2-arachidonyl glyceryl ether) induced similar neuroprotection against Aβ neurotoxicity in AD cell lines [67]. However, administration of anandamide and 2-AG in clinical trials requires additional measures and optimisations. Thus, further studies are recommended focusing the strategies to manipulate internal concentrations of endocannabinoids as therapeutics for neuroprotection in PD and AD.

In addition to the endocannabinoid neurotransmitters, the cannabinoid receptors are known to play a vital role in PD and AD pathogenesis. The CB1R antagonists, such as rimonabant and MSX-3, independently exerted neuroprotection and enhanced the dopaminergic neuronal survival [68, 69]. It is generally known that the activation of cannabinoid receptors disrupts the neuronal signalling and thus can be used as therapeutics for resting tremor, observed in PD. However, contrastingly, the aforementioned data support that the cannabinoid receptor antagonists can be developed as drugs to treat the hypokinesia, observed in PD by diminishing the CB1R activity.

Although there are only few ECS-targeted studies on Alzheimer’s disease models published in the literature, few significant data provided evidence for the potential therapeutic benefit of ECS for AD. One of the studies using AD mouse models compared the CB1R expression levels with the age and concluded that the CB1R expression levels elevate with the age [70]. This can also define as the CB1R levels are increased with the progression of the AD. Another study using AD rat models injected with Aβ to induce neurotoxicity has observed activation of CB1R to induce neuroprotective effect on hippocampus CA1 pyramidal neurons via inhibition of voltage-gated Ca^2+^ channels and suppression of Ca^2+^-activated K^+^ channels [71]. According to the data, the CB1R but not CB2R protected against electrophysiological changes such as neuronal firing frequency, induced by the injected Aβ. Therefore, although the CB1R expression is promoted in contrast to the formerly mentioned study, the activation of the receptor through CB1R agonist has exerted neuroprotection. Thus, all the aforementioned results combined emerge the necessity of understanding the interconnected pathways associated with ECS and its components for the development of precise drug targets.

In addition to the endocannabinoid neurotransmitters, the phytocannabinoids act as ligands to the ECS receptors. Among the nearly 60 different cannabinoids identified in the plant *Cannabis sativa*, cannabidiol (CBD) [72] and delta-9-tetrahydrocannabinoid (Δ^9^-THC) [73] have been studied intensely for its activity upon PD and AD models in controlling both motor and non-motor symptoms.

A study conducted in the early 1980s with THC-treated reserpine (anti-hypertensive drug)–induced rats evidenced that, though the THC alone does not induce hypokinesia, the THC-reserpine combination produced 20-fold greater hypokinesia [74]. Therefore, the authors warn the excessive use of THC compounds as drug therapeutics for PD. However, recent studies using cell cultures and primate models show positive effects of these external cannabinoids for PD. The PD-induced SH-SY5Y cell cultures were treated with THC and observed direct neuroprotective effect [75]. A research used PD models of marmoset monkeys to treat with THC and observed restoration of the locomotor activities to nearly pre-disease level [73]. The authors hypothesised that this positive effect is due to elevated expression of CB1R in the marmoset PD models. This theory can be supported and validated with the data from a previous study using marmoset models where the PD induced with MPTP, and the animals showed higher concentrations of CB1R expressed in the basal ganglia [76].

Majority of studies attempting to identify the correlation between CBD and PD have moved to clinical trials using animal models for behavioural studies [77] and PD patients [78, 79]. However, no studies following long-term effects of CBD administration were identified, nor the studies show permanent cease of neurodegeneration. Therefore, this brings back to the idea that, simultaneously, the knowledge of molecular mechanism is essential in development of therapeutics for effective prevention or cease the neurodegeneration as well as repair process. A study conducted with PC12 cell lines with MPP^+^ induced for PD, when treated with CBD, showed decreased caspase-3 activity thus increases cell viability and also protects against the MPP^+^-induced cell differentiation inhibition [80]. The study repeated for SH-SY5Y cell lines did not show a similar response to CBD treatment; thus, the authors conclude that the latter may not be a suitable model for the determination of neuroprotective effect of CBD on PD [80]. However, since the SH-SY5Y cell line has shown positive effects for THC, further trials are needed to confirm the suitability of the cell line for CBD based studies.

A recent study of transgenic Tg_4–42_ mice expressing human Aβ_4–42_ treated with Δ^9^-THC showed reduced neuronal loss when compared with the controls [81]. Furthermore, a study used a N2a-variant of amyloid β precursor (AβPP) cells treated with THC concluded with a neuroprotective effect exerted on the cells as the THC could significantly decrease the Aβ levels in the cell [82]. The authors also convey that the response is time-dependent and that repeated treatments gradually decrease the Aβ synthesis. However, in the study, the THC was administered only twice over the period of 48 h, thus may not have been sufficient to establish a conclusion. Further studies are needed to determine the dose-dependency and time-dependency of potential treatment of cannabinoids to exert a continuous neuroprotective effect and to understand the subsequent ceasing neurodegeneration and neuronal repair. Another study used AD mice models treated with cannabidiol (CBD) over a period of 7 days and observed a significant reduction of Aβ protein expression [83]. Furthermore, a study treated AD mice with CBD daily over the period of 8 months and concluded with contrasting observations that CBD had no effect on soluble and insoluble Aβ_40_ or Aβ_42_ in the cortex and in the hippocampus [84]. These contradictory arguments bring back the need of understanding the precise action of the cannabinoids in the brain prior to therapeutic development and to localise a precise unique target for neurodegeneration. The study, however, describes CBD as a potential drug for long-term preventative therapy for AD as the data showed that the CBD treatment had a positive impact on social withdrawal and facial recognition deficits in AD [84].

Even though the cellular and non-human primate models have shown positive effects for neurodegeneration, the precise molecular mechanism is not well understood. The literature evidenced that the combination of THC-CBD has shown better neuroprotective effect compared with the individual administration of the two compounds [85, 86]. However, the dose-dependency and time-dependency as well as the precise ratio of the safe administrable THC to CBD require the understanding of the molecular mechanism prior to initiating clinical studies. The long-term effects of administrating external compounds such as THC or CBD against natural components, anandamide and 2-AG, are not studied in depth. This identifies the research gap of limited longitudinal observational studies for phytocannabinoids used in PD- and AD-based clinical trials.

This trend of using phytocannabinoids as drug therapeutics for NDDs is followed by the development of synthetic cannabinoids, such as HU-210, JWH-018, and JWH-073 [87]. These synthetic cannabinoid receptor agonists (SCRAs) act on CB1R of the ECS as full agonists and consist mainly of THC, the psychoactive compound rather than CBD, which diminishes the psychoactive properties of THC [87]. As the authors review, initially, the SCRAs have become popular for recreational use as they are not detected by drug testing methods [87]. However, simultaneously, the synthetically developed cannabinoid-based drugs have exerted enhanced positive effects on PD and AD symptom modulation [88]. Further molecular and biochemical studies are essential in determining the potential side effects, especially the long-term adverse effects of the synthetic cannabinoids as drug therapeutics for NDDs. The use of high doses of THC-containing synthetic cannabinoids to act on the ECS component CB1R and its adverse effects are not elucidated. Therefore, the knowledge and understanding of the above will be essential in drug development, especially to avoid neurotoxicity in the neurodegenerated brain.

## Regulation of ECS Gene Expression

The use of the plant *Cannabis sativa* for medicinal as well as recreational purpose was not uncommon since the early Egyptian era, which was later introduced to Europe and further [59]. However, the emerging adverse effects overtime drew the usage of the plant extracts back, followed by the discovery of the endogenous system of cannabinoids in mammalian brain and its components provoking new hopes in using the ECS as a target for neurological disorders. Pharmacological manipulation and potential of regulation of the gene expression of ECS components provide a neuroprotective effect [89], making the ECS neurobiochemistry a popular target in neurodegenerative disease therapeutics. This has opened up many research threads of attempting to identify the molecular mechanisms, cascade pathways, and genetic and transcription factors involved in ECS in PD and AD.

A study evidenced that the CB1R expression was reduced in PD patients; however, it did not show whether this expression level changes were due to the parkinsonism or the l-DOPA treatment of the patients [90]. Furthermore, Parkinson’s disease rat models with reserpine treatment commonly used for decelerating the activity of the nervous system showed reduced expression of CB1R in the striatum [69]. The close association of the locomotor symptoms of PD and the ECS neurotransmitters, anandamide and 2-AG, was explained. The levels of dopamine receptor agonists used for motor symptoms significantly reduce the levels of both endocannabinoid neurotransmitters and administration of CBR1 antagonist along with the dopamine receptor agonists, which completely restored the motor symptoms of Parkinson’s disease mouse [91]. Modulation of the expression of *ABHD6* has been popular in the last few years due to its major role in ECS. Female hormones, oestrogen and progesterone, overexpressed *ABHD6* in immune cells leading to development of autoimmune reactions [92]. ABHD6 modulation of 2-AG levels, and the subsequent activation of cannabinoid receptors [93], can be utilised as a target for drug designing in PD and AD models.

## Neuroinflammation and the Endocannabinoid System

The action of inflammation in the onset of PD and AD is being evidenced through several studies [4]. As the inflammatory modulator in the brain, microglia are known to play an important role in the neuro-inflammation-mediated neurodegeneration. A recent review well describes the three major functions of microglia: constant sensing the environmental change, basic housekeeping to maintain regular neuronal function, and neuroprotective function [94]. The authors further described the microglia as a ‘double-edged sword’, due to its activity of Aβ uptake and clearance inducing neuroprotection [95], yet the persistent accumulation of Aβ activates microglia resulting in further accumulation of the peptides [94]. However, a transcriptome-sequence analysis identifies this activity to be performed by a unique type of novel microglia known as disease-associated microglia (DAM), which are located near the Aβ plaques in the AD brain [96]. It is hypothesised that these microglia act in a similar way in PD brain with Lewy body formation with α-synuclein. Even though the exact mechanism is not well understood, it is suggested that microglia activate in early PD brain in order to remove progressively accumulating α-synuclein; however, with the progression of the disease, the microglia act as phagocytes damaging the neurons [97].

The relationship between the ECS and NDDs has been studied intensively with regard to the neuroinflammation perspective. However, the effects of ECS on microglia in neuroinflammation are not well established and the associated data are limited. Several recent studies have discussed the association between the endocannabinoid system and the microglial phenotype in neuropathology. The current knowledge suggests that microglia in normal healthy brain maintain the homeostasis with M0-type morphology. The activation and polarisation of microglia due to an injury or related stimulus induce the secretion of pro-inflammatory cytokines for neuroinflammation with classical M1-type morphology. However, with the persistent activation, thus the persistent neuroinflammation leads to neurodegeneration. Alternatively, the microglia induce neuroprotection through alternative M2-type morphology by secreting anti-inflammatory cytokines [98, 99]. Similar to neurons, microglia consist of complete ECS with endocannabinoids synthesised within the cells and the GPCR cannabinoid receptors expressed on the cell membrane. Studies suggest that immune-modulatory CB2R are comparatively more abundant in microglia than CB1R and significantly upregulate during pathological conditions [100], and binding of either endocannabinoids or phytocannabinoids induces gene expression to shift microglia into less harmful M2 phenotype for neuronal protection. Researchers hypothesise that the cannabinoid agonist–initiated signalling drives the microglia into a more balanced state between M1 and M2 types and also upregulates M0-type morphology gene expression [98]. This supports the idea of therapeutic potential of both endocannabinoids and phytocannabinoids targeting microglia for neuroprotection and possible prevention of neurodegeneration strategies. However, further in-depth transcriptomics studies are necessary to determine the precise molecular mechanism of action for cannabinoids’ effect on microglia.

The inhibition of FAAH reduced the dopaminergic neuronal death, simultaneously decreasing immune-reactivity through microglial inhibition leading to improvement in parkinsonism-like motor symptoms [101]. The microglia overexpressed the CB2R and FAAH inducing inflammatory response in the brain leading to AD and CB2R deficiency reduced neuroinflammation [102, 103]. Furthermore, there was an induced expression of CB2R in activated microglia during neuroinflammation, which was absent in the inactive microglia at rest [104, 105]. The data can be supported by the study, where they used CB2R+/+ and CB2R−/− microglia cell cultures to stimulate with pro-/anti-inflammatory stimulus and observed that cells with no CB2R responded weakly to the pro-inflammatory stimuli than microglia expressing CB2R [103]. Similar results were obtained with the APP/S1 AD mouse models with further reduced concentrations of soluble Aβ in the mouse brain with no CB2R expression [103]. These data indicate the importance of the cannabinoid receptor expression for microglia activation and neuroinflammation. This suggests the potential of controlling the cannabinoid receptor expression or receptor stimulation which can be directed to inhibition of the microglia activation, and subsequent neuroinflammation and neurodegeneration. A study supported this theory that stimulation of CB1R and CB2R with receptor agonist WIN-55212-2 reduced the microglial activation [106]. Therefore, further studies shall provide clear understanding of the detailed association of ECS in microglia activation. Currently, no microglia-mediated neuroinflammation-targeted cannabinoid drugs are available or under clinical investigation; thus, it can be suggested as a novel theme in the industry to develop ECS-targeted therapeutics for neurodegeneration.

In contrast to the microglia-mediated neuroinflammation, the association of cytokines in neuroinflammation leading to neurodegeneration is well studied. A study conducted with cultured mouse macrophages concluded that 2-AG inhibits production of IL-6 and increases iNOS-dependent nitric oxide (NO) synthesis, leading to macrophage inhibition; thus, the ECS activation reduces inflammatory response [107]. Since the first discovery of expression level changes in cytokines such as IFN-α/β with THC treatment in late 1900s [108, 109], different cannabinoid-based drugs have been developed for a wide scope of diseases from general obesity to different cancers [110]. A study using AD mouse models treated with CBD observed direct impact on Aβ-mediated neuroinflammation [83]. Even though the exact molecular mechanism is unknown, the researchers suggest that the CBD may be responsible for diminishing gliosis which ultimately results in hold in neuroinflammation and neurodegeneration. However, the precise mechanism of inflammatory mediator cytokines related to endocannabinoid system in PD and AD is yet to be understood.

## Endocannabinoid System in Other Diseases

In addition to PD and AD, the ECS has been intensively studied as high potential target for therapeutics for other disease conditions as well. The involvement of ECS in pain perception and reward processing makes the ECS a target for treatment for chronic pain [111]. The ECS genetics have shown close association with energy and glucose metabolism, thereby affecting the diabetes conditions [112]. The published literature also evidence that the ECS exerts a neuroprotective effect on stroke where CB1R and CB2R antagonists have the potential to be developed into therapeutics for stroke [113]. A summary of collection of published data for other diseases associated with ECS component is given in Table 1.

**Table 1:** Published literature for the association of ECS and phytocannabinoids as potential drug target for common non-communicable diseases

Condition	Endocannabinoid ligand/receptor	Action	Reference
Other neurological disorders			
Chronic pain	Anandamide	Release of anandamide in pain suppressor neural circuits as a treatment for pain	[114]
Neuropathic pain	CB1R and CB2R	Neuronal injury and neuropathic pain reduced CB1R expression and increased CB2R expression.	[115]
Huntington’s disease	CB1R	CB1R loss is observed and overexpression exerted neuroprotection against Huntington. Therapeutic delay of CB1R loss may delay the disease progression.	[116–118]
Schizophrenia	CBD and anandamide	CBD significantly enhanced anandamide levels in serum, diminishing psychosis. Therapeutics to retain anandamide in serum could induce anti-psychotic effects.	[119–121]
Amyotrophic lateral sclerosis (ALS)	Δ^9^THC and CBD/ CB2R	Exerted neuroprotection through upregulation of CB2R in Δ^9^THC and CBD-treated ALS mouse models.	[122]
	Δ^9^THC	Delayed motor impairments and exerted neuroprotection through reducing oxidative damage to the spinal cord in THC-treated ALS mouse models.	[123]
Cardiovascular diseases			
Stroke	2-AG	Administration 2-AG to rat models significantly reduced blood flow to the brain. Authors state warning in clinical trials.	[124]
Reperfused acute myocardial infarction (AMI)	CBD	CBD-treated AMI rabbit models showed increased systolic wall thickness with increased blood flow and reduced inflammation.	[125]
Cardiac ischaemia	CBD	Reduced systolic blood pressure in patients	[126]
Diabetes			
Type-II diabetes	CBD	CBD treatment in mice significantly reduced pro-inflammatory cytokines: IFN-γ, TFN-α. TH1 cytokines were reduced and TH2 cytokines (IL-4, IL-10) were increased. Overall diabetes incidences were reduced.	[127]
	CBD and Δ^9^-tetrahydrocannabivarin (THCV)	Patients administered with CBD reduced resistin and increased glucose-dependent insulinotropic peptide levels; components directly associated with obesity. THCV administration significantly reduced fasting blood glucose levels.	[128]
Autoimmune disorders			
Corneal hyperalgesia	Δ^8^THC/CB1R	Stimulation and activation of CB1R with Δ^8^THC significantly reduced pain and corneal inflammation	[129]
Inflammatory skin disorders	CBD	Patients of psoriasis and atopic dermatitis tropically administrated with CBD ointment showed significant improvements in skin condition.	[130]

## *Cannabis sativa* and Other Phytocannabinoids

Apart from these natural cannabinoids in the brain, the CB1R and CB2R act as receptors for several external cannabinoids ligands. The plant *Cannabis sativa* (commonly known as marijuana) contains over 60 different pharmacologically active cannabinoids (phytocannabinoids) [88, 131] identified to date, out of which are Δ^9^THC, the major psychoactive compound of the plant [132], and CBD, the non-psychoactive compound of the plant [72] which are being studied widely for their neuroprotective characteristics. The phytocannabinoids for PD and AD are being used in cell culture models [75, 82], animal models [73, 133], and in clinical trials [134] in published data, as were described above. Alternatively to *C. sativa*, several other plant species have been identified for cannabinoid-like natural compounds (also known as cannabimimetics), which exert similar physiological activities. The alkylamides from *Echinacea* species (cornflower), such as *E. purpurea* and *E. angustifolia*, are structurally closely similar to the endocannabinoids allowing them to bind with cannabinoid receptors [135, 136]. The cannabimimetics have also been identified in *Theobroma cacao* (chocolate), *Heliopsis helianthoides* (oxeye), *Helichrysum umbraculigerum* (sunflower), *Acmella oleracea* (electric daisy), *Radula marginata* (liverwort), and *Piper nigrum* (black pepper) plants [137], making the CB receptor ligands naturally abundant for therapeutics.

## Limitations and Benefits of Cannabinoid-Based Drugs

Clinical trials and cohort studies using *Cannabis* plant extracts or direct administration of dried plant leaves have shown severe adverse effects on PD and AD patients, including fatal effects due to the toxicity exerted in the neurodegenerated brain [134], which raises a controversial argument of using the compounds as clinical therapeutics. This has directed researchers to study ECS and its natural components, cannabinoid receptors and ligands (anandamides and 2-AG), at the molecular and biochemical levels in order to understand its effects on neurodegenerated brain and overall on the central nervous system [48, 138].

With the understanding of the demand for further research, new techniques are being developed. A recent such technique identifies the endocannabinoids in the blood plasma through ultra-high-performance liquid chromatography-tandem mass spectrometry (UHPLC-MS/MS) [139]. Molecular docking studies for cannabinoid-based drug designing have been conducted with several bioinformatics tools [140]. These include different lead molecules, cannabinoids, and cannabimimetics [141] targeting CB1R [142] and CB2R [143, 144]. However, no successfully completed PD- or AD-targeted cannabinoid-based drug designing studies were found in the literature. This identifies the research gap of identifying potential gene targets in endocannabinoid system and to develop potential candidate leads focused on PD and AD conditions.

With the aforementioned data discussed, it must also highlight that the cannabinoid-based therapeutic drugs may have their own pros and cons. With cultural beliefs of the developing world and different perspectives, there can be a hesitation in acceptance of cannabinoids for treatment purpose. On the other hand, the administration of cannabinoid drugs must be highly regulated to avoid adverse effects of potential overdosing as well as the treatments should be well monitored by a health professional, to avoid development of addiction to the drugs. On the contrary, the ECS is a novel system still under investigation that may have a wide network associated with other primary systems of the human body. Therefore, there may be potential therapeutic benefit for wide range of non-communicable diseases. The knowledge of genetics of ECS is not well established, where the application advanced technologies such as GWAS and epigenetics, and there may lie answers to many key questions in drug development not only for NDDs but also for other disease conditions.

## Conclusion

Both PD and AD are known to have multifactor aetiology, yet the precise involvement of genetic, environmental, and behavioural factors is to be elucidated. It is well evidenced that research studies thus far have mainly focused on clinical and epidemiological cohort studies rather than on molecular-based investigations. Therefore, molecular approaches show a high demand and significance to determine the genetics and other aspects of endocannabinoid system involved in the onset of PD and AD for development of therapeutics drugs targeting preventing, ceasing, and repairing neurodegeneration.
